# Ordinary-Portland-cement solidification of Cs-137 contaminated electric arc furnace dust from steel production industry in Thailand

**DOI:** 10.1016/j.heliyon.2024.e25792

**Published:** 2024-02-03

**Authors:** Klitsadee Yubonmhat, Pattaranipa Gunhakoon, Poonnaphob Sopapan, Nikom Prasertchiewchan, Witsanu Katekaew

**Affiliations:** a Radioactive Waste Management Center, Thailand Institute of Nuclear Technology, Nakhon Nayok, 26120, Thailand

**Keywords:** EAFD, Cs-137, Radwaste, Accidental melting, Conditioning, Leaching

## Abstract

The cementation was used to immobilize the Cs-137 contaminated electric arc furnace dust (EAFD). Various mixing recipes were used to prepare the EAFD-cement waste form specimens. The strength test, the ANSI/ANS-16.1 leaching test and the immersion test were performed to judge whether the cured specimens satisfy the Radwaste disposal requirements. The strengths of all specimens were higher than the acceptable limit (3.45 MPa). The specimen's strength depended on the EAFD content, the water-to-binders ratio, and the curing time. Moreover, it could be affected by the leaching of the cement and EAFD components. The leaching results showed that the Cs-137 could be totally leached after ending of the test. There is a positive correlation between the quantity of Cs-137 in the leachate and the leachate pH. The Cs-137 leachability index (LI) decreased as the EAFD content and the ratio increased. The LI values ranged from 5.9 to 6.4. The Cs-137 leaching from the specimens could be controlled by the diffusion or the surface wash-off, depending on the recipes used. Additionally, the Cs-137 leaching might be controlled by multiple mechanisms. The findings reasonably recommend that the recipe with the ratio of 0.40 and 40 % EAFD could be used for the EAFD immobilizing.

## Highlights

• The strength of the EAFD waste forms depended on the EAFD content, the water-to-binders ratio, and the curing time.
• The strength of the waste forms decreased as a result of the OPC and EAFD components being leached.
• After the leaching test was ended, the Cs-137 could be completely leached from the waste forms.
• The higher the Cs-137 in leachate, the higher the leachate pH.
• The Cs-137 leaching from the waste forms could be controlled by the diffusion or the surface wash-off.

## 1 Introduction

Steel could be the backbone of the development of modern industrial economy because the production of important facilities (e.g., automobile, ship, machinery, and tools) needs steel. This powerful material can be produced via three different routes, the open-hearth furnace (OHF) route (≈0.4 %), basic oxygen furnace (BOF) route (≈75 %), and electric arc furnace (EAF) route (≈25 %) [[1], [2], [3]]. The OHF process is in clear decline and is being replaced by the BOF and EAF processes [1] because of its environmental and economic disadvantages [3,4]. At present, the use of the EAF process appears to be more popular than the BOF process [1]. That could be because of its lower energy consumption and carbon emissions, compared to the BOF process [3,4].

Electric arc furnace dust (EAFD) is a by-product generated during EAF steel production and is collected in the baghouse filter system [5]. Making one ton of steel can generate about 15–20 kg of EAFD [6,7]. This dust contains heavy metals (e.g., Zn, Fe, Cd, Pb, and Cr) [[8], [9], [10], [11]] and its composition depends on the input materials used in the production process [5]. With the presence of these metal elements, EAFD is therefore considered officially hazardous in many countries (e.g., Brazil, USA, Germany, and Turkey) [8,12], including Thailand according to Notification of Ministry of Industry on List of Hazardous Substances B.E. 2556 (2013) [13]. Safe and proper management of this dangerous dust is then desired.

There are three main choices for dealing with EAFD, landfill disposal, recycling, and reusing for other purposes. The cost for the first choice is quite high because it is necessary to meet greater requirements and also needs the control of disposal sites [5,6,14]. This high cost is then the motivating factor for EAFD recycling and reusing. Many researchers have attempted to recover valuable elements (e.g., Zn, Fe, and Pb) from EAFD [6,[15], [16], [17]]. EAFD was also used as, for instance, a solid catalyst in biodiesel production [18], a raw material in the production of decorative ceramic glazes [10,19], and an additive to cement-based mixtures for civil construction [11,20,21].

Unfortunately, radioactively contaminated EAFD cannot be treated using the mentioned methods due to specific reasons regarding legal control. Radioactive EAFD could be resulted from melting of radioactive sources inadvertently included in the input raw materials (e.g., steel scrap) and from melting of the radioactively contaminated raw materials, during EAF steel production process [2,22]. Depending on the regulations that each country has in place for its steel manufacturers, radiation detection systems for the input raw materials might not be required. Therefore, radioactive materials (e.g., radioactive sources that escape from regulatory control, and radioactively contaminated metal) cannot be detected and then they can be introduced into EAF.

Metal, slag and EAFD are the output products generated from the EAF steel production process [23]. Hence, they can be contaminated with radioactive contaminants. The contaminants are distributed differently in these products because of their physical and chemical properties [2]. There has been reported that cesium-137 (Cs-137) is mainly distributed into EAFD with the distribution factor of 0.5–1, while the factors of distribution into slag and metal are 0.1–0.5 and 0.001, respectively [24]. These factor values are also consistent with the Cs-137 distribution results reported in Ref. [25]. With the assumption that when Cs-137 sources are under high temperatures for producing steel, the Cs-137 is in the form of gas and it interacts with oxygen to form cesium superoxide (CsO_2_). The superoxide is then transported with the generated off-gas [32] and EAFD. For this reason, the EAFD is therefore contaminated with Cs-137.

In 2018, around 880 tons of radioactive EAFD were found in the steel production factory in Thailand and the EAFD was contaminated with Cs-137 in high activity concentrations ranging from 420 to 486,680 Bq/kg [26]. These Cs-137 activity levels are extremely high in comparison with those of the Croatian (6.83–20.9 Bq/kg) EAFD [2], and the Canadian EAFD (555–1110 Bq/kg) [27]. In Thailand, the Cs-137 EAFD is considered as radioactive waste and classified as low-level radioactive waste (LLW) [26,28,29]. As a result, such radioactive dust must be controlled by the Nuclear Energy for Peace Act [30,31] and managed according to its supporting regulation, the Ministerial Regulation on Radioactive Waste Management, B.E. 2561, 2018 [29].

In Thailand, radioactive waste containing artificial radionuclides (e.g., Cs-137) has not been allowed to be disposed of in any sites [29]. However, there are two permitted methods that might be possible to manage this dust (i.e., treatment, and conditioning). A recent attempt has been made to utilize the leaching process to reduce the activity level of the EAFD and reuse the Cs-137 leached in the preparation of ^137^Cs/^137m^Ba generators [32]. Moreover, the author research group is now performing a series of lab-scale experiments (e.g., EAFD washing, Cs-137 removal by co-precipitation process, and EAFD conditioning) so as to find out a suitable approach for managing this dust waste [26,[33], [34], [35]]. Immobilization of the Cs-137 EAFD in cement matrix is the focus of this work because of its low cost and simple operation [36,37].

The choice of a conditioning option depends on the type of radioactive waste. Vitrification is the technique suitable for stabilizing and encapsulating high-level radioactive waste (HLW) [38]. In the vitrification process, HLW is mixed with glasses under high temperatures (≈1000 ^๐^C) [39]. Bituminization is one of the conditioning techniques. Radioactive waste is embedded in molten bitumen under the temperature (softening point) of up to 132 ^๐^C [40]. The technique is normally used for conditioning LLW and intermediate-level radioactive waste (ILW) [41,42], and particularly suitable for water-soluble radioactive waste [40]. Cementation is also extensively used for immobilizing LLW and ILW [43,44]. In the cementation process, radioactive waste is solidified using ordinary Portland cement (OPC) as the primary binder [36]. Since the Cs-137 EAFD is the LLW, bituminization and cementation are then the applicable techniques for the EAFD conditioning.

Immobilization of the waste in suitable matrices can significantly reduce the potential for radionuclide release into the environment. The use of bitumen has the advantages over cement, such as higher waste loading, and better resistance to leaching of radionuclides [40,42]. It was also reported that the use of bituminization for the waste conditioning leads to an immobilized waste that is about 5 times smaller in volume than that obtained by cementation [42]. However, the combustibility of bitumen can lead to potential fire hazard caused by an accidental fire, resulting in the radionuclide release into the atmosphere. Furthermore, bitumen solidified waste exhibits the lower strength, radiation durability and resistance to biodegradation, compared to cement solidified waste [40]. It is obvious that bituminization is more complicated than cementation because it needs higher operation temperatures, while cementation can be operated under room temperatures. As these reasons, cementation is then the most widely applicable technique used for conditioning LLW and ILW.

Cementation has been employed for conditioning various radioactive waste, such as, the metal oxide waste [44], the incineration ash [43], the spent radioactive liquid scintillator [45] and the concrete waste [46]. In general and also in these researches, the qualification tests such as the compressive strength, leaching, and immersion tests on the obtained solidified wastes are performed to judge whether the waste forms meet the waste form requirements for disposal of radioactive waste [47]. Although cement has been used for solidification of several radioactive wastes, no Cs-137 contaminated EAFD has been conditioned using cement. Small amount of radioactive EAFD was added to the immobilization mortar of the containers of low- and medium-activity repositories [48]. However, to date, the EAFD immobilization for the disposal purpose has not been studied.

Therefore, the objective of the study is to study the cement solidification of the Cs-137 EAFD so as to evaluate whether the EAFD waste forms will be disposed of safely. The EAFD-OPC samples (waste forms) were prepared by various formulations. The authors investigated the effects of EAFD addition and water-to-binders ratio on the behaviour (i.e., leachability index, and controlling leaching mechanism) of Cs-137 leaching from the samples. Here, the ANSI/ANS-16.1 leching test method was used to study the behaviour. The compressive strength test was also conducted on (1) 28-day cured specimens, (2) 90-day cured specimens, (3) specimens subjected to the leaching test (unconventional test added by the authors), and (4) specimens subjected to 90-day immersion test. The pH of the leachates from the immersion and leaching tests was also measured.

## 2 Materials and methods

### 2.1 Raw materials

The primary binder used is the OPC produced by TPI Polene (Public) Co., Ltd. It was purchased from the local store. The Cs-137 EAFD was received from the steel production factory and used as an additive binder. Bulk density of the OPC and the EAFD is approximately 1.22 g/cm^3^ and 0.88 g/cm^3^, respectively. The chemical compositions of these binders were determined using X-ray fluorescence (XRF), as given in Table 1. The major oxides of the OPC are SiO_2_, and CaO. In contrast, the main oxides of the EAFD are ZnO, and Fe_2_O_3_.

**Table 1:** Chemical composition of the OPC and EAFD.

Compound	OPC (wt.%)	EAFD (wt.%)
ZnO	0.03	34.17
Fe_2_O_3_	2.68	33.63
Na_2_O	0.37	7.03
Cl	0.22	4.41
SiO_2_	21.78	3.65
CaO	63.42	3.16
MgO	2.16	2.68
SO_3_	3.45	2.63
K_2_O	0.46	2.50
MnO	0.05	2.40
PbO	–	1.72
Al_2_O_3_	4.96	0.71
Cr_2_O_3_	–	0.40
P_2_O_5_	0.10	0.29
CuO	0.02	0.24
Br	–	0.20
TiO_2_	0.25	0.08
SnO_2_	–	0.06
SrO	0.03	–
Others	0.02	0.04

**Fig. 1:**
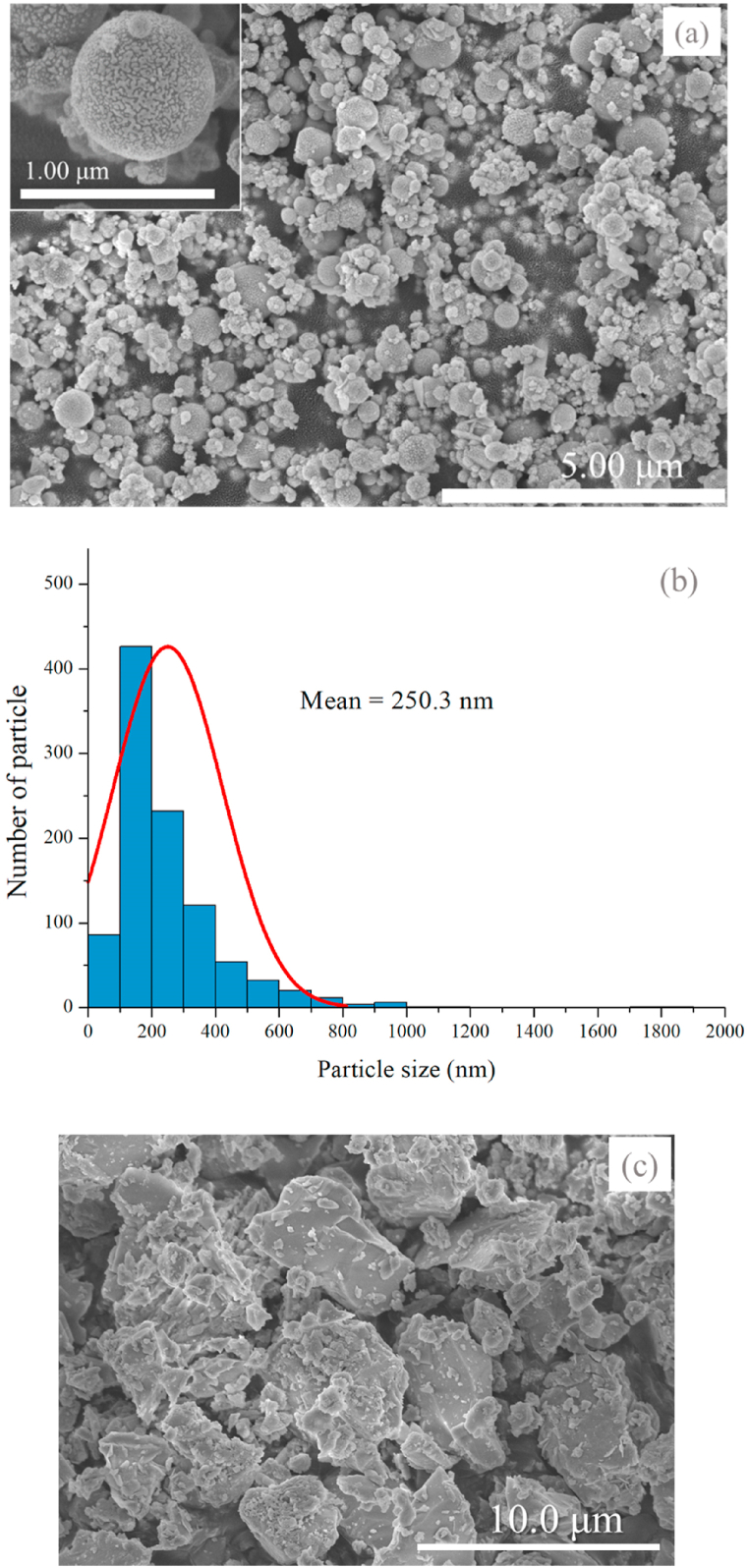
(a) FESEM micrograph of the EAFD, (b) the EAFD particle size distribution, and (c) FESEM micrograph of as-received OPC.

The microstructure of the EAFD was also observed under field emission scanning electron microscopy (FESEM), as shown in Fig. 1(a), along with the EAFD particle size distribution shown in Fig. 1(b). The EAFD is spherical particles. The particle size ranges from 31.7 to 1865.9 nm, with the average size of 250.3 nm. The FESEM micrograph of the OPC can be seen in Fig. 1(c). The OPC grains have smooth surfaces and are nonuniformly shaped.

### 2.2 EAFD-OPC waste form preparation

**Fig. 2:**
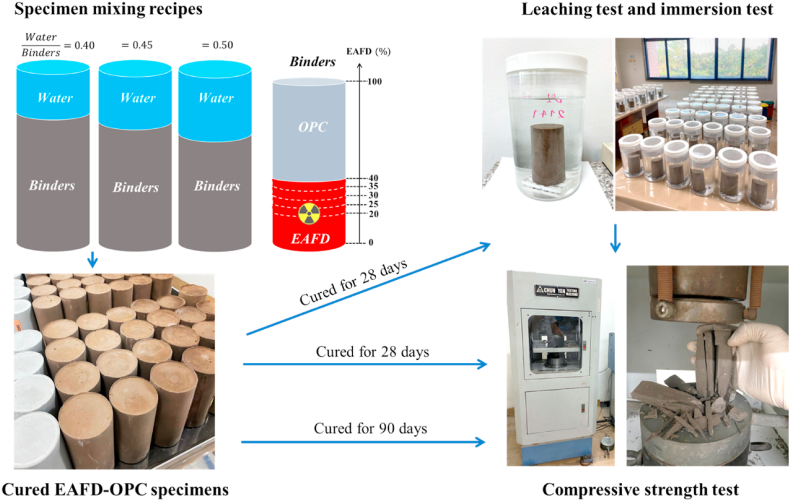
A process for qualification tests of the EAFD-OPC waste form specimens.

The EAFD-OPC waste form specimens were prepared by mixing the two binders with different EAFD contents at water-to-binders (W/B) ratio of 0.40, 0.45 and 0.50. Here, the EAFD content was set to be 0, 20, 25, 30, 35, and 40 wt% of the binders' total weight. This maximum EAFD content was set based on the previous work [35], which found that when EAFD contents exceed 40 wt%, the prepared EAFD-OPC specimens' strength was below the acceptable limit (3.45 MPa) [47]. Firstly, the EAFD and the OPC were blended into a bowl-lift stand mixer at a 166 rpm for 5 min. Then, tap water with required amount was added to the mixture and blended for another 5 min at the same speed. At the end of mixing, small amount of the EAFD-OPC paste was collected and cured for 28 days. The FESEM with energy dispersive spectroscopy (EDS) and a High Purity Germanium (HPGE) gamma spectrometer were used to analyze the cured paste's elements and activity concentration, respectively. The remaining paste was poured into the cylindrical polyethylene moulds (10-cm height and 5-cm diameter). The paste was poured in each mould in two approximately equal layers. Each time the paste was poured, hand compaction (rodding) and vibration by hands were performed to obtain a better compaction of the paste and remove air voids in the paste. After that, the moulds were closed with their lids and allowed to cure in air (ambient temperature = 33.1 ± 1.6 °C) for 28 or 90 days, depending on the following tests. After the end of curing, the obtained waste form specimens were removed from the moulds. The dimension and mass of the samples were measured. Then, all samples were subjected to the tests. The whole process for all tests is depicted in Fig. 2.

To analyze the total Cs-137 activity of a waste form, the dried sample was ground and 10 g of the ground sample was digested using clean-off liquid concrete remover. The digested sample was mixed with tap water to obtain the total volume of a liter of liquid state sample. The liquid sample was poured into a Marinelli beaker (model 138-G) and then analyzed its Cs-137 activity concentration using the HPGE gamma spectrometer. Here, the certified reference material for radioactivity measurements in water sample (model code: EG-ML), manufactured by Eckert & Ziegler isotope products, CA USA, was used for the calibration. This reference material is in the same type of breaker used for the liquid sample. For each mixing recipe, three replicated liquid samples were made. The averaged result was used for calculating the total activity of each EAFD-OPC waste form prepared.

### 2.3 Compressive strength test

The compressive strength test was performed on the prepared specimens using a testing machine (Chun Yen, CY-6690) shown in Fig. 2. All specimens were tested without capping. In fact, the compressive strength test on the 28-day and 90-day cured specimens was performed before conducting the other tests (i.e., immersion and leaching tests). This is for the screening purpose. The strength values of the specimens should be ≥ 3.45 MPa, as recommended by U.S. Nuclear Regulatory Commission [47]. To replicate the experiment, four specimens prepared with the same recipe were tested and the results were expressed as mean ± the sample standard deviation (SSD). This test was also performed on the samples subjected to the leaching and immersion tests described in the following section.

### 2.4 Leaching test and immersion test

Each studied case, three specimens cured for 28 days were subjected to the leaching test. The ANSI/ANS-16.1-2003 standard test [49] was applied to evaluate the leaching of Cs-137 from all studied specimens. Each specimen was placed in a container containing the leaching solution of tap water (pH = 7.93 ± 0.14), as shown in Fig. 2. The leachant volume is 10 times the surface area of the specimen. The leachate was changed and sampled after cumulative leaching times of 2 h, 7 h, 1 day, 2 days, 3 days, 4 days, 5 days, 19 days, 47 days, and 90 days. The gamma spectrometer was employed to measure the activity of the Cs-137 in all sampled leachates, and the obtained result was then used to determine the total activity of each leachate. The pH of each leachate was also measured. After ending of the test, the compressive strength of all samples was measured using the testing machine.

For immersion test, three 28-day cured samples from each studied recipe were submerged in tap water for total immersion time of 90 days, no replacement of leaching solution is made. The experimental setup is the same as that of the leaching test. During the immersion test, the pH of each immersion solution was measured after the same cumulative leaching times as in the leaching test. After ending of the test, the strength test was performed on these specimens tested.

### 2.5 Analysis of leachability index and controlling leaching mechanism

During the leaching test, Cs-137 radionuclides in a specimen tested can be leached continuously to leaching solution. The cumulative fraction leached (CFL) from the specimen is defined as [49]:

(1) $$CFL=\frac{\sum a_{n}}{A_{0}}$$

where a_n_ is the total activity of the Cs-137 released from the specimen during leaching interval n, $\sum a_{n}$ is the cumulative activity of the Cs-137 released from the specimen from the beginning of the test to the end of the leaching interval of concern, and A_0_ represents the total activity of the Cs-137 in the specimen at the beginning of the test.

The leaching rates observed with the specimens are most often explained by radionuclide diffusion. If < 20 % of the Cs-137 is leached from the specimen (i.e., CFL <0.20), the effective diffusivity D is calculated using the following equation [49].

(2) $$D=\pi {\left[\frac{a_{n}/A_{0}}{{\left(\Delta t\right)}_{n}}\right]}^{2}{\left[\frac{V}{S}\right]}^{2}T$$

where V is the volume of the specimen, S is the surface area of the specimen, (Δt)_n_ is the duration of the nth leaching interval, and T is the mean time of the leaching interval, expressed as [49]:

(3) $$T={\left[\frac{\sqrt{t_{n}}+\sqrt{t_{n-1}}}{2}\right]}^{2}$$

where t_n_ is the cumulative leaching time at the end of the current interval, and t_n-1_ is the cumulative leaching time at the end of the previous interval.

If ≥ 20 % of the Cs-137 is leached from the specimen (i.e., CFL ≥0.20), the D can be derived from the specimen diameter (d) and the dimensionless time factor (G). The factor is a function of the CFL and is provided in Ref. [49]. In this case, the D can be figured out using the following equation.

(4) $$D=\frac{Gd^{2}}{t}$$

where t is the elapsed time from the beginning of the test to the end of the current interval.

The leachability index (LI) is the parameter used to evaluate the release of contaminants due to diffusion mechanism. It is recommended that the LI for each leaching interval should be > 6.0 [47]. The LI is expressed as [49]:

(5) $$LI=\log_{10}\left(\frac{\beta }{D}\right)$$

where β is a constant value of 1.0 cm^2^/s.

However, the contaminant leaching involves not only diffusion but also the surface wash-off and the dissolution. The release of radio-contaminants from a waste form can be due to one or superimposition of these three mechanisms [37]. The exchange kinetics between the leaching solution and the waste form surface results in the contaminant leaching from a waste form. The leching rate is linearly dependent on the concentration of the contaminant. A first-order reaction model [37] can be used to describe this behavior. It is assumed that the leaching process is controlled by the surface wash-off mechanism [50]. The contaminant leaching from most cement-based waste forms is controlled by diffusion [51]. The diffusion process controlling the leaching obeys Fick's laws [43]. If the leaching contaminant is a major component of a waste form, its release into leaching solution results in structural damage to the waste form. This leaching is assumed to be a dissolution-controlled process [43,50]. In the present study, the most widely used method [43,52,53] was conducted so as to determine the controlling leaching mechanism. It was simply determined by considering the slope of the linear relationship between the logarithm of the CFL and the logarithm of the cumulative leaching time.

## 3 Results and discussion

### 3.1 FESEM-EDS analysis of samples

**Fig. 3:**
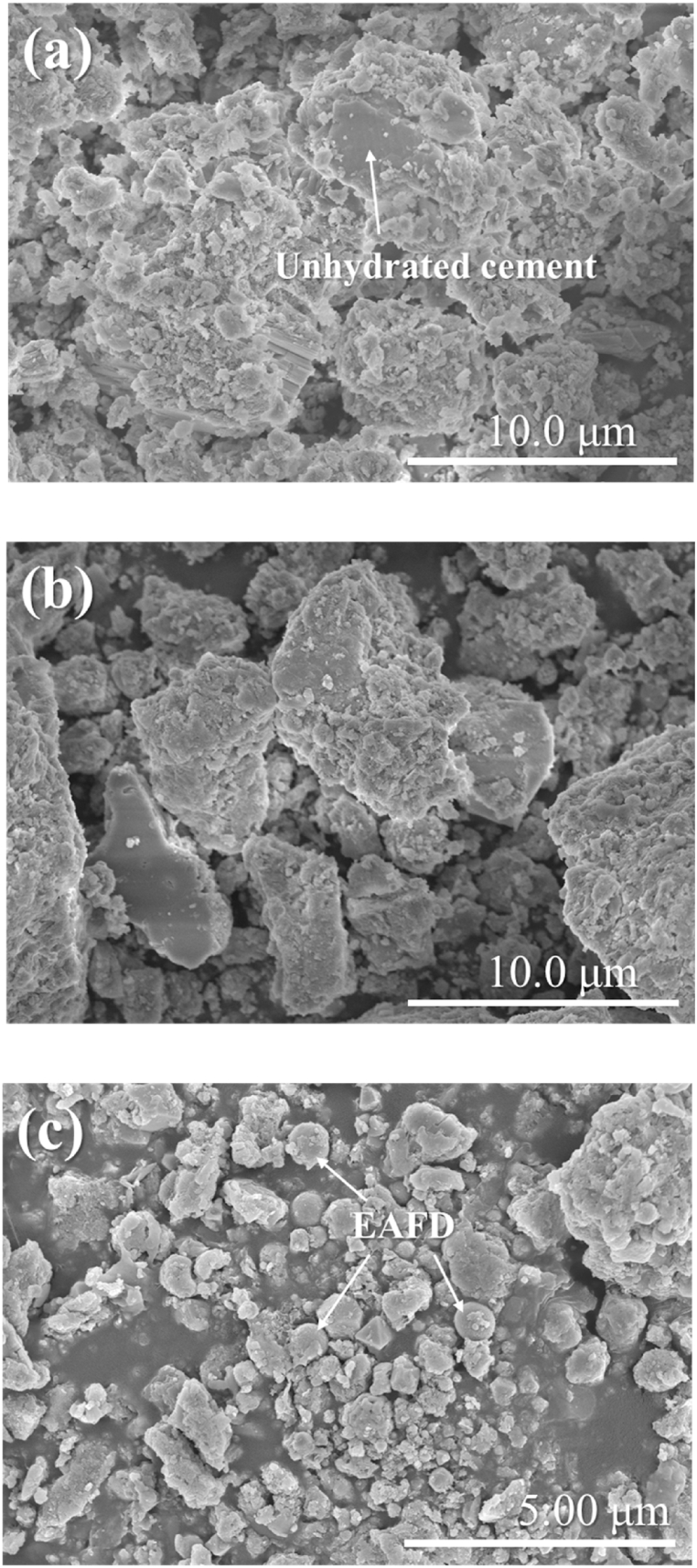
FESEM images of (a) cured OPC without EAFD, and (b, c) cured EAFD-mixed OPC at different magnifications.

The waste form specimens were prepared using various recipes and the used ingredients were the OPC, EAFD and tap water. As shown in Fig. 1(c), the OPC grains have smooth surfaces. Due to cement hydration, the surface of the OPC grains is obviously changed, as shown in Fig. 3(a). The hydrated cement particles have rough surfaces. This is probably due to the inner- and outer-hydration products (high- and low-density calcium silicate hydrate gels, and calcium hydroxide) deposited on the surfaces of unhydrated OPC grains [54,55]. In contrast, the surface of the grains is rougher when there is an addition of EAFD, as shown in Fig. 3(b). Fig. 3(c) clearly reveals the presence of EAFD particles incorporated in cement.

**Fig. 4:**
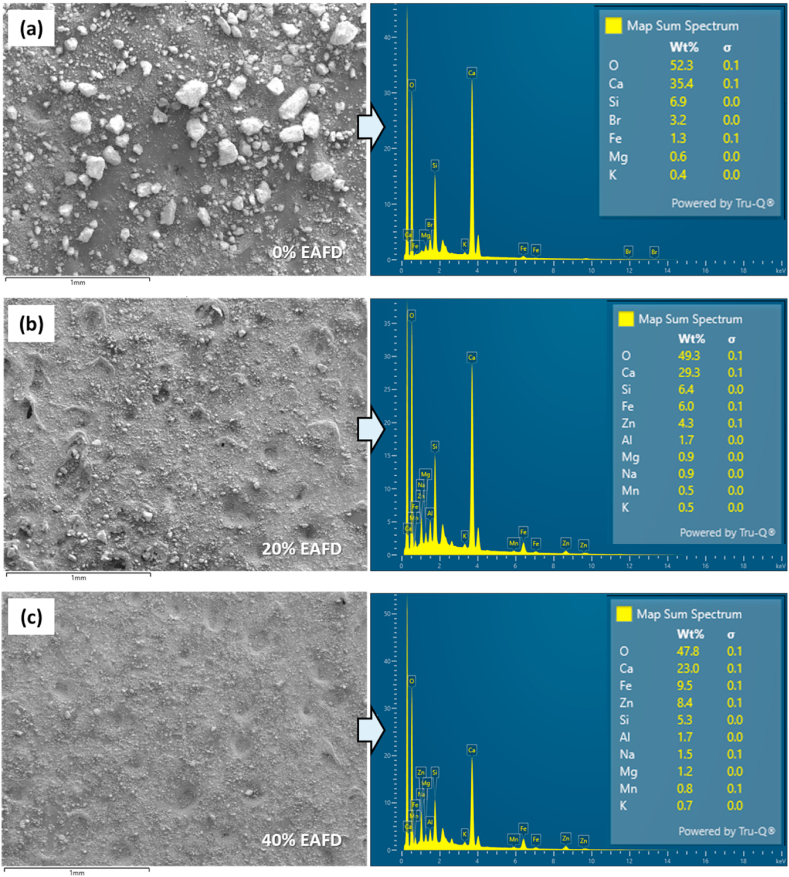
FESEM images and energy dispersive spectra of the 28-day cured EAFD-OPC samples containing (a) 0 % EAFD, (b) 20 % EAFD, and (c) 40 % EAFD. The W/B is 0.45.

The 28-day old pastes were also analyzed its elements using FESEM with EDS and the results are illustrated in Fig. 4. The EDS analysis confirms the identity of dominant element as detected by XRF (Table 1). The hydrated cement without EAFD consists mainly of Ca, Si and Br. However, Br that is not found in the as-received OPC can be detected. It was reported that Br can be found in water [56]. As a result, it is reasonable to assume that the water used to prepare the samples contains the Br that was detected. The cement samples with EAFD compose of the elements that found in both OPC and EAFD. The major elements in these samples were Ca, Si, Fe and Zn. It is clearly shown that Ca and Si significantly reduce with an increasing in EAFD content because the OPC was replaced by the EAFD, as per the recipes. In contrast, the dominant elements of EAFD (i.e., Fe and Zn) increase with an increase of the EAFD content used for replacing OPC.

### 3.2 Compressive strength

After the compression test, the columnar-type failure [57] occurred in all specimens tested, as illustrated in Fig. 2. Frictional forces generated between the machine loading plates and the two contact faces of a specimen tested result in a variety of specimen failure patterns. If the force is reduced, the columnar type of failure can be expected [58]. Here, the occurrence of this failure pattern could be attributed to the absence of capping pads and retainers commonly used for distributing load while the specimen contact faces are not flat, resulting in decreasing the force.

**Fig. 5:**
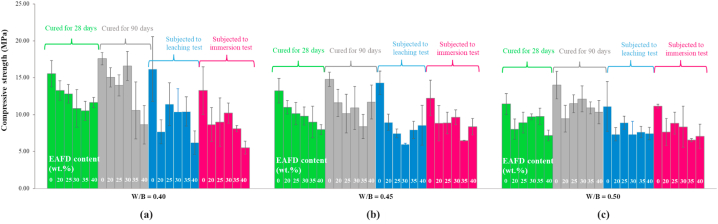
Compressive strength of the EAFD-OPC waste form specimens prepared with different EAFD contents at different water-to-binders ratios: (a) 0.40, (b) 0.45, and (c) 0.50.

The results of the compressive strength test as a function of the EAFD content and W/B ratio are shown in Fig. 5. The measured strength of all samples meets the requirement (i.e., compressive strength ≥3.45 MPa) [47]. Hence, in term of compressive strength, all the studied recipes can be potentially used for conditioning the Cs-137 EAFD to be disposed of in authorized sites. The lowest strength value is 5.52 ± 0.88 MPa, which is of the 40%EAFD-0.40 W/B specimens subjected to the immersion test. The highest value is up to 17.57 ± 0.83 MPa, which is of the 0%EAFD-0.40 W/B specimens cured for 90 days. It is clearly shown that the strength shows a decreasing trend with increasing the water-to-binders ratio, and this strength behavior is also consistent with the behavior of the cement-ash materials reported in Ref. [43]. This behavior can be attributed to the specimen porosity. When the ratio increases, the specimen porosity also increases [59], resulting in the decline of specimen strength [35]. Moreover, it is obviously indicated that adding EAFD reduces the strength of the specimens for all studied cases. The similar study [35] indicated that when EAFD is mixed with OPC, the EAFD addition leads to an increase of the specimen porosity, and the specimen strength is inversely proportional to the porosity. This could be the reason why compressive strength of all specimens with EAFD is lower than that of the control specimens (0 % EAFD).

By comparing the two different curing times, one can observe that the 90-day-old specimens with EAFD have a higher compressive strength than the 28-day-old specimens with EAFD. This can be attributed to the presence of ZnO (the main oxide of the EAFD, see Table 1) in the specimen, which it act as a retarder in setting time of cement [60]. Hence, it is necessary for EAFD-OPC conditioning to cure the hardened Cs-137 EAFD for up to 90 days to achieve the higher hardness.

The samples exposed to the wet conditions showed exhibited lower compressive strength, compared to both 28-day-old and 90-day-old samples. The constituent stability influences the cement-based material's durability in aggressive environment (e.g., wet condition). Here, the decrease in the strength after exposing to tap water for 90 days could have resulted from the leaching of the specimen constituents (i.e., OPC elements/hydration products, and EAFD elements).

**Fig. 6:**
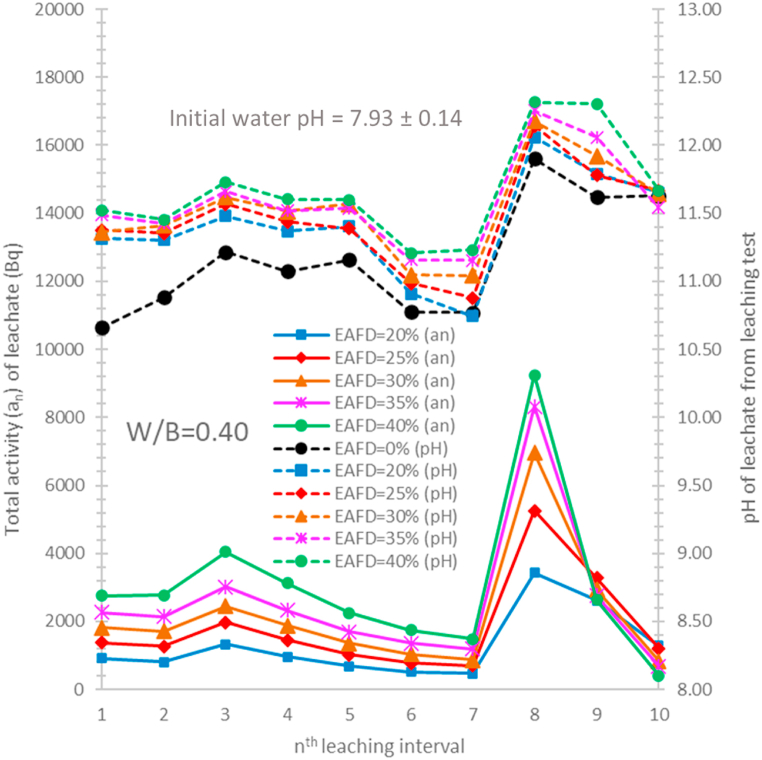
The total activity and pH of the leachates from the leaching test for different EAFD-OPC specimens prepared with water-to-binders ratio of 0.40.

**Fig. 7:**
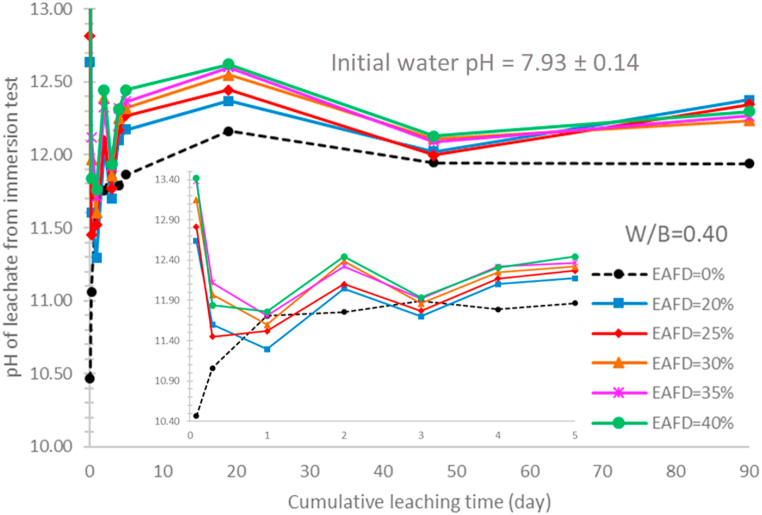
pH of the leachates from the immersion test for different EAFD-OPC specimens prepared with water-to-binders ratio of 0.40.

As shown in Table 1, Calcium oxide (CaO) is the main component of the OPC. This oxide reacts with water, generating calcium hydroxide [Ca(OH)_2_] and creating a high-alkaline solution (pH ~ 12.4) [61]. As exposed to deionized water, there was the dissolution of the hydration products [54,55] (calcium hydroxide, and calcium silicate hydrate gels) of cement-based materials and the products can be leached from the materials [62,63]. It was reported that the calcium ion leaching increases the porosity of the materials, decreasing compressive strength and durability of the materials [35,62,64]. The pH values of the leachates for the specimens without EAFD provide evidence of the existence of the hydration product leaching, as illustrated in Fig. 6, Fig. 7. The pH of all leachates is much higher than the initial pH of tap water used (7.93 ± 0.14). As shown in Fig. 6 (specimens without EAFD), the leachate pH for all test interval shows that hydration products can be leached continuously to the leaching solution at varying rates throughout the leaching test. Moreover, the pH results from the immersion test also confirms the continuous leaching of the hydration products, as illustrated in Fig. 7 (specimens without EAFD). The leachate pH rapidly increased during the early period of the test, slightly increased until 19 days, and then slightly decreased until the end of the test. The supply and consumption of the hydration product (i.e., calcium hydroxide) in the leaching solution was reported to be the cause of the pH change in the leachate [63]. As a result, the pH decline after 19 days is probably caused by an imbalance between calcium hydroxide consumption and supply.

As represented in Fig. 6, Fig. 7, the pH of the leachates for the specimens with EAFD is higher than that of the leachates for the specimens without EAFD. This indicates that the leachate pH is influenced not only by the quantity of the hydration products leached but also by the quantity of the EAFD elements (e.g., heavy metals) that can be released to leaching solution, generating alkaline solution [65,66]. Interestingly, the leachate pH is closely related to the total Cs-137 quantity leached, as illustrated in Fig. 6. In this work, the Cs-137 is one of the EAFD elements. In the EAF steel production process, this radioactive element is in the form of cesium superoxide (CsO_2_) that readily reacts with water to form cesium hydroxide (CsOH), causing alkaline solution [32]. Consequently, the leachate's alkalinity is the consequence of the presence of the EAFD Cs-137 in the leachate.

As shown in Fig. 5, in each recipe studied, it was found that the strength of the specimens subjected to the leaching test is quite the same as that of the specimens subjected to the immersion test, especially in the case of the 0.50 W/B. This could be because the same test duration (90 days) was used and there was no leachate saturation effect on the leaching of the specimen constituents during the immersion test, which caused the specimen strength to decrease [64].

### 3.3 Characteristics, parameters and mechanisms of Cs-137 leaching

**Fig. 8:**
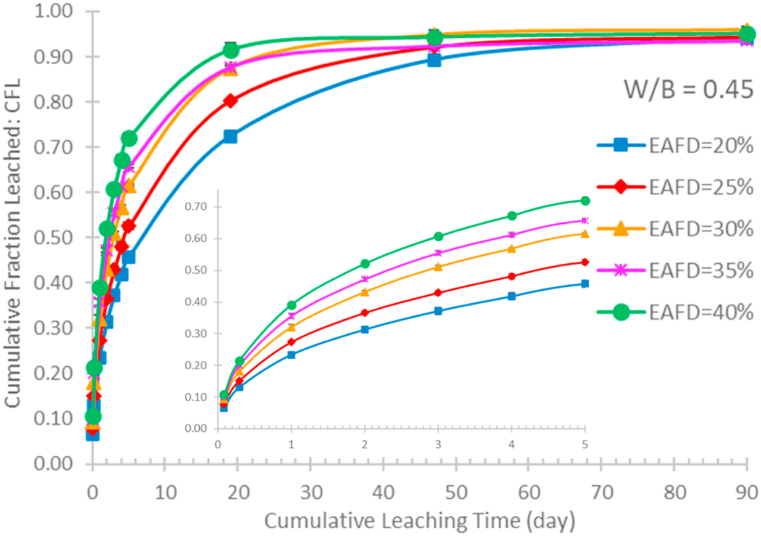
Cumulative fraction leached as a function of cumulative leaching time for different EAFD-OPC specimens prepared with water-to-binders ratio of 0.45.

The behaviour of the Cs-137 leaching from the EAFD-OPC specimens prepared with various recipes were studied using the ANSI/ANS-16.1 leching test method. The total activity values illustrated in Fig. 6 were used to calculate the CFL using equation (1). In each interval, the CFL values obtained from three identically prepared specimens were averaged and reported as mean ± SSD. Fig. 8 illustrates the plots of the average CFL as a function of cumulative leaching time. Obviously, the addition of higher EAFD content results in a higher rate of the Cs-137 leaching. At the cumulative time of 90 days, it can be seen that the CFL value for each specimen almost equals to 1. According to equation (1), this indicates that the Cs-137 radionuclides have been completely leached from the specimen after the end of the test, as $\sum a_{n}$ equals to A_0_.

The effective diffusivity (D) was calculated using equation (2) or (4). The mean time T required for calculating the D value shown in equation (2) was computed using equation (3). The obtained D values were used to figure out the leachability index (LI) according to equation (5), which is one of the parameters used to judge whether the waste forms meet the requirements for radioactive waste disposal. The average LI, as computed in accordance with the ANSI/ANS-16.1 procedure, should be higher than 6.0 [47]. Table 2 shows the average LI of Cs-137 for all cases studied. The LI shows a decreasing trend with increasing the EAFD content and water-to-binders ratio. The obtained LI values indicate that all studied recipes prepared with a water-to-binders ratio of 0.40 can be used for immobilizing the EAFD containing Cs-137. In contrast, the immobilization recipes containing 30 %, 35 % and 40 % EAFD and prepared with a water-to-binders ratio of 0.45 or 0.50 cannot be efficient because the LI values are below or equal to 6.0.

**Table 2:** The Cs-137 leachability index, and the data of the linear relationship between log(cumulative fraction leached) and log(cumulative leaching time).

W/B ratio	EAFD content (%)	LI ± SSD	Slope	Coefficient of determination, R^2^	Controlling leaching mechanism
0.40	20	6.3 ± 0.1	0.38	0.98	diffusion
	25	6.4 ± 0.1	0.37	0.98	diffusion
	30	6.2 ± 0.1	0.36	0.97	diffusion
	35	6.1 ± 0.1	0.35	0.96	diffusion
	40	6.1 ± 0.1	0.35	0.94	diffusion
0.45	20	6.3 ± 0.1	0.38	0.97	diffusion
	25	6.2 ± 0.1	0.36	0.95	diffusion
	30	6.0 ± 0.2	0.34	0.93	surface wash-off
	35	6.0 ± 0.2	0.32	0.90	surface wash-off
	40	5.9 ± 0.3	0.31	0.88	surface wash-off
0.50	20	6.2 ± 0.1	0.35	0.96	diffusion
	25	6.2 ± 0.2	0.33	0.93	surface wash-off
	30	6.0 ± 0.3	0.30	0.89	surface wash-off
	35	5.9 ± 0.3	0.28	0.85	surface wash-off
	40	5.9 ± 0.4	0.27	0.82	surface wash-off

**Fig. 9:**
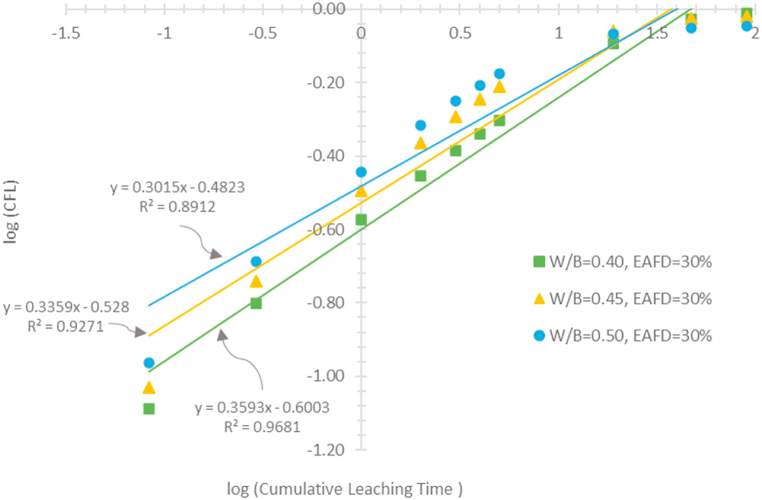
Logarithm of the Cs-137 cumulative fraction leached versus the logarithm of cumulative leaching time for examination of the leaching mechanisms.

Fig. 9 illustrates the linear graphs used for determining the mechanisms controlling the Cs-137 leaching. As reported in various researches [43,52,53], the controlling leaching mechanisms can be determined from the slope of the graph. Table 2 lists the slopes of the graphs and the corresponding determination coefficients (R^2^). If the slope <0.35, the leaching process is controlled by the surface wash-off mechanism. If the slopes ranging from 0.35 to 0.65, the diffusion is the controlling leaching mechanism. For the slope >0.65, the process can be controlled by the dissolution mechanism. According to the obtained slope values, the leaching mechanisms were then determined and summarized in Table 2. It was demonstrated that diffusion was the mechanism that controls the Cs-137 leaching from all specimens prepared with a water-to-binders ratio of 0.40. The surface wash-off was the controlling mechanism when the ratio and dust content reached the appropriated specific values. It is important to note that as the EAFD content increased, both the slope and the R^2^ decreased. This suggests that multiple mechanisms probably controlled the Cs-137 leaching. Three controlling mechanisms can be responsible for the behaviour of radionuclide leaching from a cement-based waste form. The multi-mechanism models [37] can be used to comprehend the complexity of radionuclide leaching. The utilization of the models is now beyond the authors’ knowledge. However, it is possible to conclude that the surface wash-off is the dominant mechanism controlling the Cs-137 leaching if certain values of the water-to-binders ratio and the EAFD content are reached.

It is possible to draw the conclusion that the immobilization recipe with a water-to-binders ratio of 0.40 and 40 % EAFD is most suitable for immobilizing the Cs-137-containing EAFD. This conclusion can be reasonably reached considering dust loading and the criteria for compressive strength and radionuclide leaching. The suitability of the radioactive waste forms to be disposed of is not only determined by the mechanical and leaching properties, but also other properties (e.g., thermal stability, and radiation durability) [47]. These properties need to be investigated further to ensure that the waste forms will be disposed of safely and without causing harm to the public and the environment.

## 4 Conclusions

The Cs-137 contaminated electric arc furnace dust (EAFD) from the steel production industry has been immobilized using cementation with ordinary Portland cement (OPC). The waste form specimens were prepared by blending the two binders (OPC and EAFD) with different EAFD contents (0, 20, 25, 30, 35, and 40 wt% of the binders’ total weight) at a water-to-binders ratio of 0.40, 0.45 and 0.50. The strength test, the ANSI/ANS-16.1-2003 leaching test and the 90-day immersion test were performed on the cured specimens to decide whether the specimens meet the waste form requirements for radioactive waste disposal.

The strength test was conducted on 28-day cured specimens, 90-day cured specimens, specimens subjected to the leaching test, and specimens subjected to the immersion test. The strengths of all specimens tested were found to be above the acceptable limit (3.45 MPa). The specimen's strength decreased when the ratio was increased and the OPC was partially replaced with the EAFD. This could be attributed to the specimen's increased porosity as these two factors increased. The ZnO in the EAFD could retard the cement setting time, the strength of the specimens cured for 90 days was then higher than that of the specimens cured for 28 days. The strengths of the specimens subjected to the leaching and immersion tests were nearly identical. However, compared to the specimens cured for 28 and 90 days, their strengths were lower. The reason for this is that the alkalinity of leachates provided strong evidence that OPC and EAFD components (e.g., hydration products, heavy metals, and Cs-137) can be leached from the specimens to the leachants, increasing the porosity and decreasing the strength of the specimens.

The leaching and immersion tests were performed for 90 days on the 28-day cured specimens. The leaching test results revealed that the Cs-137 could be released continuously throughout the leaching test and completely leached at the end. Interestingly, there is a correlation between the total amount of Cs-137 leached and pH of leachate; the higher the Cs-137 in leachate, the higher the leachate pH. The pH change in the leachates from the immersion test also implied that there was an imbalance between calcium hydroxide consumption and supply. The calculation of the Cs-137 leachability index (LI) was based on the diffusion mechanism, as per the leaching test. It was found that the LI showed a decreasing trend with increasing the EAFD content and the ratio. The LI values range from 5.9 to 6.4, depending on the mixing recipes used.

The mechanisms that controlled the Cs-137 leaching from the specimens were determined by examining the slopes of the linear relationship between the logarithm of the CFL and the logarithm of the cumulative leaching time. The results demonstrate that, depending on the recipes used, the Cs-137 leaching could be controlled by the diffusion or the surface wash-off. The results also imply that the multiple mechanisms might be responsible for the Cs-137 leaching from the specimens.

Based on the mechanical and leaching properties obtained, the authors suggest that the solidification recipe with a water-to-binders ratio of 0.40 and 40 % EAFD is reasonable for immobilizing the EAFD containing Cs-137.

## Data availability statement

Data included in article/supp. material/referenced in article.

## CRediT authorship contribution statement

**Klitsadee Yubonmhat:** Writing – review & editing, Writing – original draft, Methodology, Investigation, Formal analysis, Conceptualization. **Pattaranipa Gunhakoon:** Investigation. **Poonnaphob Sopapan:** Investigation. **Nikom Prasertchiewchan:** Project administration. **Witsanu Katekaew:** Resources.

## Declaration of competing interest

The authors declare that they have no known competing financial interests or personal relationships that could have appeared to influence the work reported in this paper.
